# Surgical retrieval of PICC-related right atrial thrombus in a child with acute lymphoblastic leukemia: a case report

**DOI:** 10.1186/s12872-020-01536-8

**Published:** 2020-05-27

**Authors:** Yu Lan Luo, Jinmei Zhang, Menglin Tang

**Affiliations:** Department of Pediatric Intensive Care Unit, West China Hospital, Sichuan University, Guoxuexiang 37th, 610041 Chengdu, Sichuan P.R. China

**Keywords:** Peripherally inserted central catheters, Right atrial thrombus, Acute lymphoblastic leukemia, Case report

## Abstract

**Background:**

Peripherally inserted central catheters (PICCs) are widely used in cancer patients for administering chemotherapy drugs, antibiotics, and nutrients. PICC-related thrombi are not uncommon and may result in pulmonary embolism and the formation of thrombi in the right atrium. The latter are associated with an increased risk of subsequent morbidity or mortality because of their potential for embolization in the pulmonary vasculature.

**Case presentation:**

A 16-year-old male with acute lymphoblastic leukemia (ALL) was admitted to our hospital after an echocardiographic examination revealed a ring-like structure in the right atrium that was still present after 6 months’ anticoagulation treatment with aspirin. The boy had had a PICC inserted 2 years previously for chemotherapy; the PICC was intact and successfully removed 18 months after insertion when chemotherapy is finished. Subsequent computer tomography and radiography differentiated right atrial ring-shaped mass with a diameter of approximately 15 mm. Cardiac surgery was performed to remove the mass which was found to be a calcified thrombus.

**Conclusion:**

Although this is a rare occurrence, recognition of the possibility of a calcified thrombus may minimize the misdiagnosis of PICC-related thrombus and allow surgical retrieval if the thrombus is sufficiently large.

## Background

Peripherally inserted central catheters (PICC) are widely used in patients for the administration of chemotherapy and antimicrobial drugs, parenteral nutrition, and, recently, for blood sampling, especially for cancer patients [1–3]. Complications of PICCs, including accidental removal, fracture, embolization, and migration of the tip have been reported in the literature [4, 5]. The most common complication is venous thrombosis, which can result in catheter removal, treatment interruption, and life-threatening events such as pulmonary embolism [6]. Although catheter-related thrombi in the right atrium and the pulmonary artery are rarely reported [7, 8], they may lead to serious complications and even death if the migrated thrombus is not removed in time [9]. Herein, we present a case of PICC-related thrombus in the right atrium that was successfully removed surgically.

## Case presentation

**Fig. 1:**
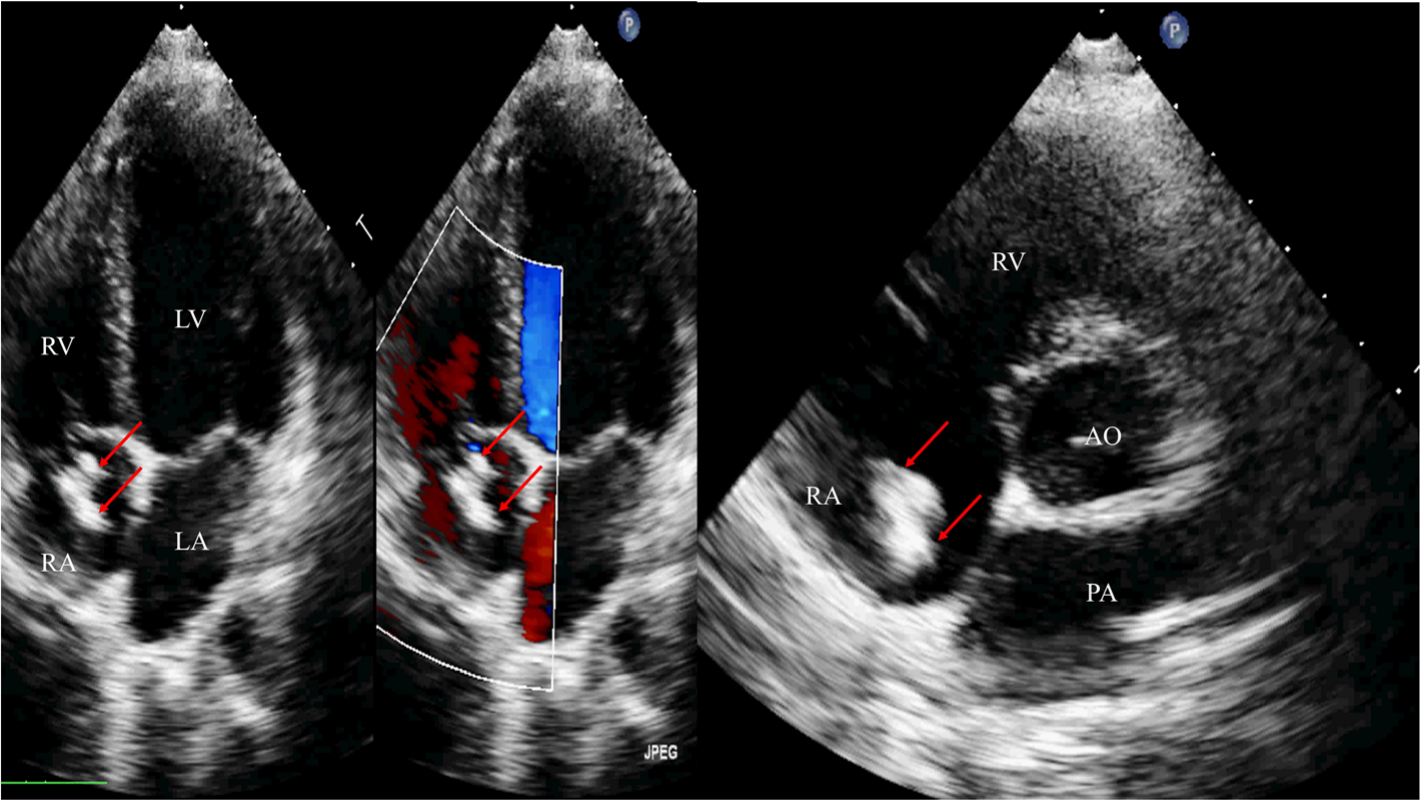
Echocardiographic imaging of the calcified thrombus. The apical four chamber view and the parasternal short axis view clearly demonstrate the large, mobile mass (arrow) originating from the free wall of the right atrium. RA—right atrium; LA—left atrium; RV—right ventricle; LV—left ventricle; Ao—aorta

A 16-year-old male with acute lymphoblastic leukemia (ALL) was admitted to our hospital because an echocardiographic examination revealed a ring-like structure in the right atrium that had been present for 6 months and there was no change of the ring-like structure after 6-month aspirin anti-coagulation therapy. Two years previously, the child had been diagnosed with ALL and a PICC had been placed for intravenous access during treatment in the general medical ward for chemotherapy and parenteral nutrition in a children’s hospital. The PICC remained intact and was successfully retrieved after 18 months of chemotherapy. A regular transthoracic echocardiography (TTE) examination revealed a large, mobile, ring-like mass originating from the free wall of the right atrium that measured 15 × 20 mm (Fig. 1a-b). The child was asymptomatic and the physical examination was also negative. When he presented to our hospital for a definite diagnosis, cardiac CT and radiography was performed to confirm the diagnosis.

**Fig. 2:**
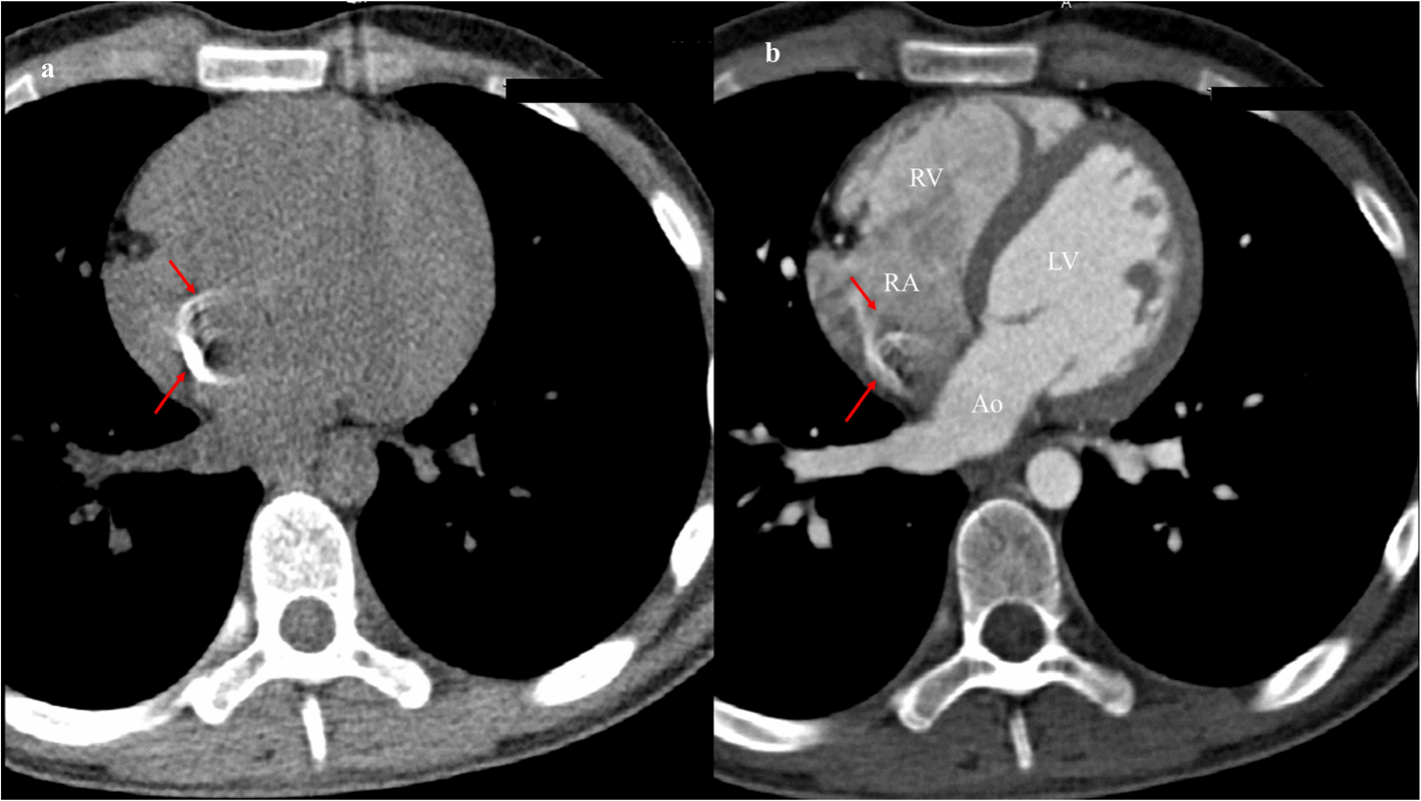
CT showed the large calcified thrombus in the right atrium

**Fig. 3:**
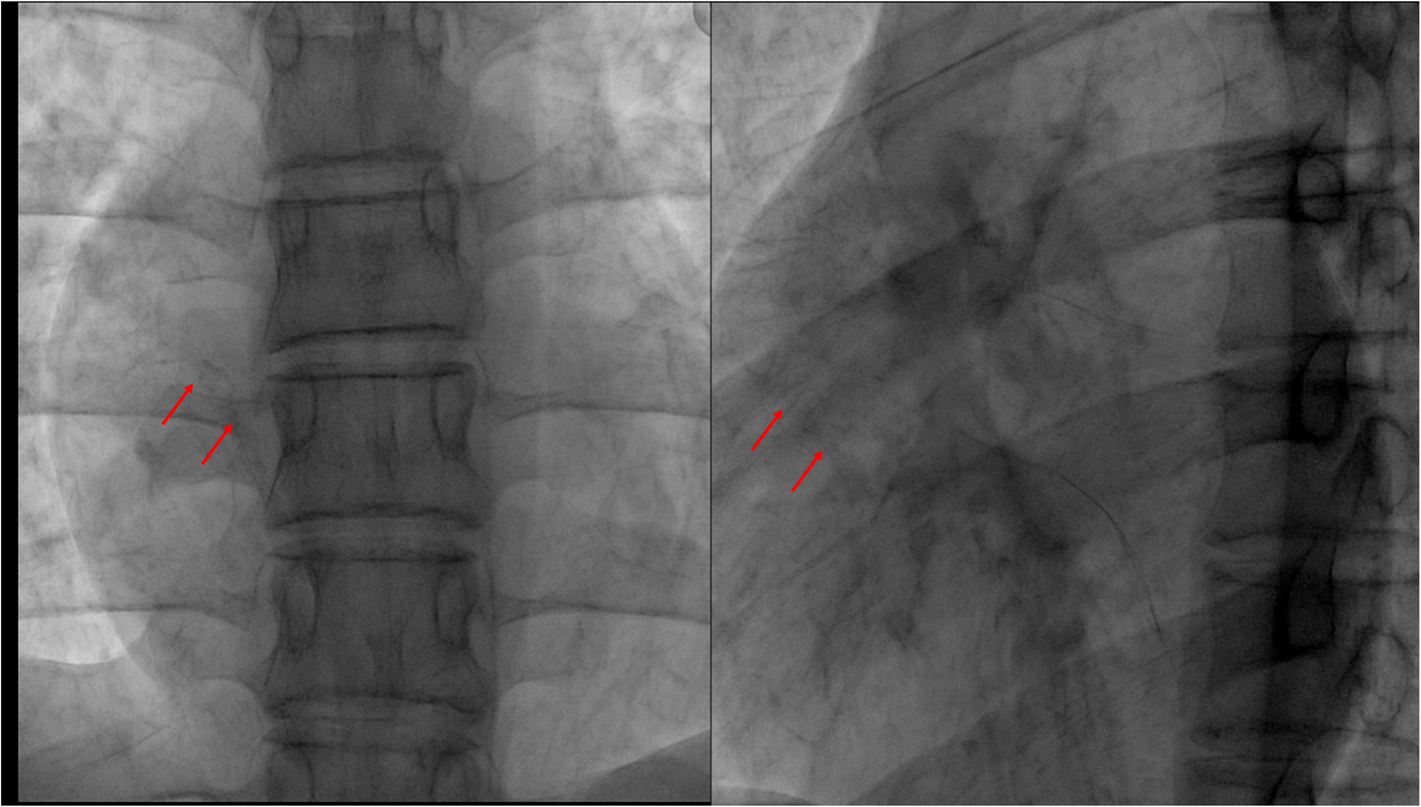
Radiology revealed the large, mobile calcified thrombus in the right atrium without PICC distal catheter

Cardiac computed tomography revealed a calcified mass with a diameter of about 15 mm in the right atrium close to the inferior vena cava (Fig. 2a-b). Radiography confirmed the diagnosis and showed the absence of a PICC distal catheter in the right atrium and also that the mass was mobile (Fig. 3a-b). Given the large size of the mass, interventional radiology was used as part of a multidisciplinary approach to determine the optimal removal strategy. It was finally decided to remove the mass surgically.

**Fig. 4:**
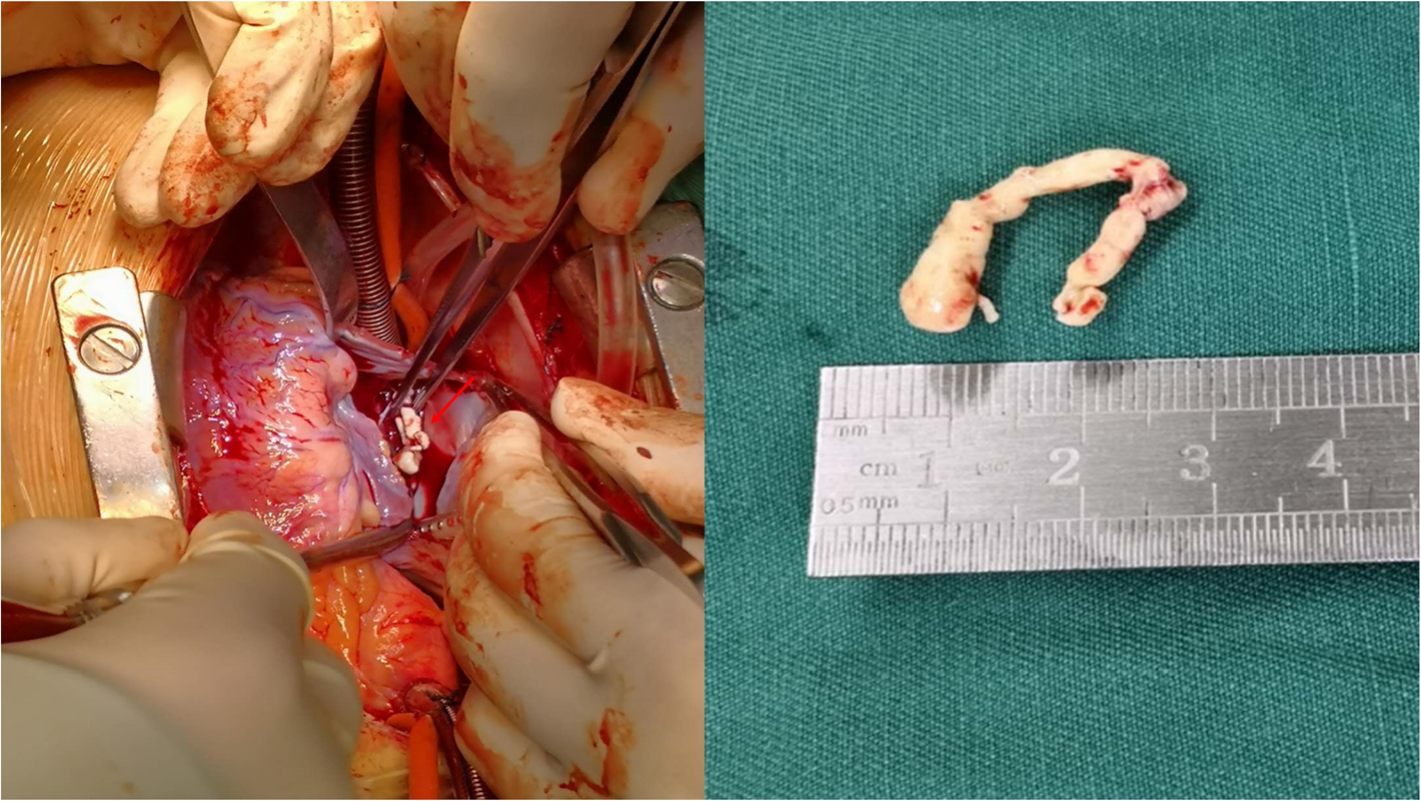
**a** Thoracotomy was performed to remove the calcified thrombus. **b** Photograph of the gross specimen showed a solid ring-like calcified thrombus which was removed from the right atrium

The patient was operated on using cardiopulmonary bypass, with aortic and bicaval cannulation performed in the standard manner. The right atrium was opened and a large, solid, ring-like mass was found to be eroding into the orifice of the inferior vena cava (IVC) (Fig. 4a-b). After the mass was removed, it was cut open, showing a 2 mm pinhole in the middle, indicating that it had formed by initial calcification around the tip of the PICC which progressed after the retrieval of the PICC.

## Discussion and conclusions

PICC has become a reliable and commonly used device for long-term intravenous therapy in different sets of patients. However, PICC-related deep vein thrombosis (DVT) can result in severe cardiovascular events such as congestion of limb vein and pulmonary thrombosis. The rate of PICC-related DVT has been found to be 2.4% in all categories of patients but was higher when onco-hematologic patients was excluded [10].

Although patients with PICCs receive regular catheter care, it is inevitable that, after a period of time, PICC-related DVT may occur in some patients. Most patients with DVT are asymptomatic [11], therefore examination by medical imaging is particularly important. The detection of PICC-related DVT is mainly based on vascular ultrasonography or echocardiography, and, if necessary, contrast-enhanced computed tomography (CT) may be used. If vascular ultrasonography or echocardiography shows a clot in a deep vein or in the right atrium, DVT often presents as a filling defect in contrast-enhanced CT [11].

Anticoagulation therapy plays a crucial role in the prophylaxis and treating of PICC-related DVT. Part of the DVT will usually resolve after several weeks of anticoagulation therapy [12], but other parts of the DVT suchas chronic right atrium thrombus and pulmonary embolism may remain unchanged. When medication is no longer effective, removal of the DVT by open heart surgery may be an effective solution. Despite the high risk of open heart surgery, it is effective for patient recovery from the consequences of PICC-related DVT. Surgical removal is an effective and safe procedure for calcified thrombus in right atrium and it is to be preferred in elective conditions especially in young asymptomatic patients without hemodynamic involvement, who are at low risk of surgery related morbidity and mortality [13, 14].

As presented in this case, the thrombus in right atrium appeared as a strong echo and high-density clot on echocardiography and CT scan respectively. The presence of these features may indicate surgical removal to resolve this type of calcified thrombus.

## Data Availability

The datasets used in the case are available from the corresponding author upon reasonable request.

## Abbreviations

PICC: Peripherally inserted central catheter
ALL: Acute lymphoblastic leukemia
TTE: Transthoracic echocardiography
IVC: Inferior vena cava
RA: Right atrium
LA: Left atrium
RV: Right ventricle
LV: Left ventricle
AO: Aorta

## References

[1] Evans RS, Sharp JH, Linford LH, Lloyd JF, Tripp JS, Jones JP, Woller SC, Stevens SM, Elliott CG, Weaver LK (2010). Risk of symptomatic DVT associated with peripherally inserted central catheters. J Chest..

[2] Sriskandarajah P, Webb K, Chisholm D, Raobaikady R, Davis K, Pepper N, Ethell ME, Potter MN, Shaw BE (2015). Retrospective cohort analysis comparing the incidence of deep vein thromboses between peripherally-inserted and long-term skin tunneled venous catheters in hemato-oncology patients. J Thromb J.

[3] Li J, Fan YY, Xin MZ, Yan J, Hu W, Huang WH, Lin XL, Qin HY (2014). A randomised, controlled trial comparing the long-term effects of peripherally inserted central catheter placement in chemotherapy patients using B-mode ultrasound with modified Seldinger technique versus blind puncture. J Eur J Oncol Nurs.

[4] Chen CC, Liang CD, Huang CF, Chung MY (2006). Percutaneous removal of a peripherally inserted central catheter remnant using cardiac catheterization. J Pediatr Int.

[5] Hashimoto Y, Fukuta T, Maruyama J, Omura H, Tanaka T (2017). Experience of peripherally inserted central venous catheter in patients with hematologic diseases. J Intern Med.

[6] Zochios V, Umar I, Simpson N, Jones N (2014). Peripherally inserted central catheter (PICC)-related thrombosis in critically ill patients. J J Vasc Access.

[7] Thanigaraj S, Panneerselvam A, Yanos J (2000). Retrieval of an IV catheter fragment from the pulmonary artery 11 years after embolization. J Chest.

[8] Liu JC, Tseng HS, Chen CY, Chern MS, Chang CY (2004). Percutaneous retrieval of 20 centrally dislodged port-a catheter fragments. J Clin Imaging.

[9] Cakir F, Geze S, Ozturk MH, Dinc H (2014). Percutaneous endovascular removal of intracardiac migrated port a catheter in a child with acute lymphoblastic leukemia. Braz J Anesthesiol.

[10] Balsorano P, Virgili G, Villa G, Pittiruti M, Romagnoli S, Gaudio A, Pinelli F (2020). Peripherally inserted central catheter-related thrombosis rate in modern vascular access era-when insertion technique matters: a systematic review and meta-analysis. J Vasc Access.

[11] Tian G, Zhu Y, Qi L, Guo F, Xu H (2010). Efficacy of multifaceted interventions in reducing complications of peripherally inserted central catheter in adult oncology patients. Support Care Cancer.

[12] Burns KE, McLaren A (2009). Catheter-related right atrial thrombus and pulmonary embolism: a case report and systematic review of the literature. Can Respir J.

[13] Spencer K, Weinert L, Pentz WH (1999). Calcified right atrial mass in a woman receiving long-term intravenous phosphate therapy. J Am Soc Echocardiogr.

[14] Fabi M, Gesuete V, Testa G, Balducci A, Picchio FM, Gargiulo G (2011). Calcified Thrombus in right atrium: rare but treatable complication of long-term indwelling central venous catheter. Cardiol Res.

